# Systematic review of prospective hazard analysis in radiation therapy

**DOI:** 10.1002/mp.18110

**Published:** 2025-09-10

**Authors:** Jonathan Hindmarsh, Sonja Dieterich, Jeremy Booth, Paul Keall

**Affiliations:** ^1^ Image X Institute Faculty of Medicine and Health University of Sydney Eveleigh New South Wales Australia; ^2^ Department of Radiation Oncology UC Davis Medical Center Sacramento California USA; ^3^ Northern Sydney Cancer Centre Royal North Shore Hospital St Leonards New South Wales Australia; ^4^ Institute of Medical Physics School of Physics University of Sydney Camperdown New South Wales Australia

**Keywords:** failure mode and effect analysis (FMEA), hazard, high‐risk failure modes, risk, safety, system theoretic process analysis (STPA), TG‐100

## Abstract

**Introduction:**

Prospective hazard analysis (PHA) was introduced to the wider medical physics community by the initiation of American association of physicists in medicine task group 100 in 2003. Since then, there has been increasing interest in the applicability of PHA to radiotherapy for the purpose of keeping patients safe and assessing the risks within the whole practice of radiotherapy. The purpose of this research was to review the PHA literature focusing on which techniques and technologies have been assessed, how they have been assessed, and what can be learnt.

**Methods:**

The search for English language, peer‐reviewed, full‐text articles was conducted across five databases and the citations of three seminal papers using a common search strategy. The collation, filtration, and analysis of articles was conducted in accordance with the preferred reporting items for systematic reviews and meta‐analyses (PRISMA) statement reporting standard utilizing the following PICOS approach: Population: x‐ray external beam radiation therapy, Intervention: prospective hazard analysis, Comparison: none, Outcome: patient safety, Study characteristics: details of applied technique.

**Results:**

689 unique studies were identified. 62 were determined to be eligible for inclusion. PHA has been applied to C‐arm treatment systems (17), stereotactic radiosurgery (8), TomoTherapy (6), stereotactic body radiotherapy (5), Ethos (5), Halcyon (3), MRIdian (3), review activities (3), commissioning (2), unity (1), volumetric modulated arc therapy (1), surface guidance (1), CyberKnife (1), RefleXion (1) and other novel software and hardware systems (6). Disciplines involved in the studies were physicists (92%), physicians (75%), radiation therapists or dosimetrists (71%), external experts (38%), and facilitators (33%). Failure mode and effects analysis (FMEA) was used in 75% of studies, 10% used FMEA derived methods, 10% used system theoretic process analysis, and 5% used other methods. From the FMEA studies, 579 high‐risk failure modes were extracted covering all aspects of the radiotherapy process, 50% applied to patient treatment delivery sessions and 25% applied to contouring and treatment planning. The mitigation strategies recommended by studies tended to add to the departmental workload.

**Conclusions:**

62 studies were identified that used PHA in radiotherapy, within the included studies: patient journey was the most analyzed process, of the disciplines physicists were involved in the most studies, FMEA the most common technique, and the delivery of patient treatment was the greatest source of high‐risk failure modes.

## 1 INTRODUCTION

Radiation therapy is a treatment technique that comes with many inherent risks, risks that for the majority of the time are well managed but have led to serious consequences for patients.^1^, ^2^, ^3^ The need to manage these risks to keep both patients and staff safe is critical and requires methods that are reproducible and easy to implement. In radiation therapy, management of risks has been achieved through consensus guidelines based on expert assessment. However, as the field moves to more complex technologies and techniques, the method of assessing and managing the risks also needs to evolve.

The most rigorous method of pre‐emptively assessing the risks within a system is prospective hazard analysis (PHA). PHA involves the use of a formalized method of assessing and/or defining a system, technology, or technique, evaluating it for hazards, prioritizing the identified hazards and reassessment following the development of mitigation strategies to reduce the risk of the identified hazards. A benefit of PHA is that it can be performed during development of a system, technology, or technique. Although prospective analysis still requires expertise and knowledge, it doesn't require years to be spent with the system to fully understand its workings prior to generating safe working practices and QA programs. Within radiation therapy, PHA can be used to assess the risks of bespoke technologies, allow site‐specific assessment of risks, and facilitate cost/benefit analyses to assist in optimally deploying the limited resources available for risk mitigation.

PHA has been in use since at least the early 1960s,^4^, ^5^ when the United States Department of Defense used it to assess the reliability of systems. Failure modes and effect analysis (FMEA) was one of the first techniques developed. In 2003, the American association of physicists in medicine (AAPM) commissioned Task Group 100^6^ (TG‐100) to investigate the use of PHA in radiation therapy (RT). In doing so they introduced the wider medical physics community to the concept of PHA. In the literature the effect of the formation of this task group is observable when in 2007 the first study explicitly applying a PHA technique in radiation oncology was published.^7^

In addition to introducing the concept of PHA and the FMEA technique to the broader RT community, the aims of TG‐100 were twofold: (1) equip individual groups to create their own quality assurance (QA) process (including where the clinical experience to develop consensus guidelines that include QA recommendations does not yet exist), and (2) to make existing QA processes more efficient by focusing efforts on failure modes deemed high‐risk.

Although there have been many publications on PHA in radiotherapy, there has not been a systematic review to synthesize the publications and to generate broad learnings for the entire community. To address this knowledge gap, here the authors systematically review the use of PHA in x‐ray external beam radiation therapy to determine which techniques and technologies have been assessed, how they have been assessed and what can be learnt from the published literature.

## 2 METHODS

### 2.1 Search strategy

Database searches were made on the Web of Science, SCOPUS, MEDLINE, PubMed, and Embase databases. The search strategy across all databases was for the terms ‘hazard analysis’, ‘process analysis’, ‘hazard assessment’ OR (‘failure modes’ AND ‘error’) within the title, abstract or keywords of texts that also included ‘radiation therapy’ or ‘radiotherapy’. To reduce the initial number of articles, those that mentioned ‘chemotherapy’ or ‘surgery’ in the abstract, title or keywords or had ‘survival’ as a keyword were excluded. Citation searches were performed for three seminal papers, TG‐100^6^ and two other studies^8^, ^9^ selected for their early publication and high citation count. In each case, articles referencing ‘brachytherapy’ or ‘proton therapy’ were excluded along with any non‐peer reviewed studies.

Through the process of designing the search strategy, it was found that while ‘risk analysis’ is widely used, it was not used in literature separate from the terms that were included in the search criteria. Conversely, “hazard analysis” did identify additional references not otherwise captured. Regarding the omission of acronyms, with most journals requiring the full spelling of acronyms in the abstract, it was felt that the search strategy was sufficient to capture the relevant papers. Where it was identified that studies were missing from the search, they were added manually.

The references captured in the searches were exported to Covidence,^10^ a web‐based collaboration software platform that streamlines the production of systematic and other literature reviews. Covidence was used to exclude duplicate studies and to assist in the assessment of whether studies met the selection criteria (Table 1).

**TABLE 1:** Population, intervention, comparison, outcome, and study characteristics (PICOS) criteria for the literature review.

Include	Exclude
**Population**	
Linear accelerator‐based x‐ray external beam radiation therapy	Brachytherapy Intraoperative RT Co‐60 Electrons Non‐human Non‐therapy
**Intervention/Exposure**	
Prospective hazard analysis	
**Comparator/Context**	
Patient safety context	
**Outcome**	
Patient safety recommendations	
**Study characteristics**	
Studies must include details of the applied analysis, such as but not limited to: ◦ System description ◦ Identified hazards ◦ Recommendations resulting from analysis	
**Other**	
English language Peer reviewed full text articles	Review articles, conference abstracts, proceedings, book chapters, and editorials Articles where the full text can't be found Articles without a DOI

### 2.2 Study selection and data extraction

Following recommended guidelines, one reviewer reviewed the title and abstract of all the studies, and two other reviewers performed a second independent review of the title and abstract to assess if they met the study selection criteria (Table 1). If there was a disagreement in assessment, the studies were moved to full‐text review. One reviewer then performed full‐text review of the remaining studies and data extraction. The following data were extracted from each study:

1. Study information (title, lead author, year, and country)
2. Aim of study
3. Target system including starting and end point for the analysis
4. Details of team performing the analysis
5. Involvement of a facilitator and if yes, their background
6. Timeframe of analysis
7. Details of the hazard analysis technique
8. Details of the system description
9. Number of identified hazards
10. Rating method and threshold for high‐risk hazards
11. Number and details of the high‐risk hazards
12. Number and details of the recommendations

## 3 RESULTS

### 3.1 Study selection

**FIGURE 1:**
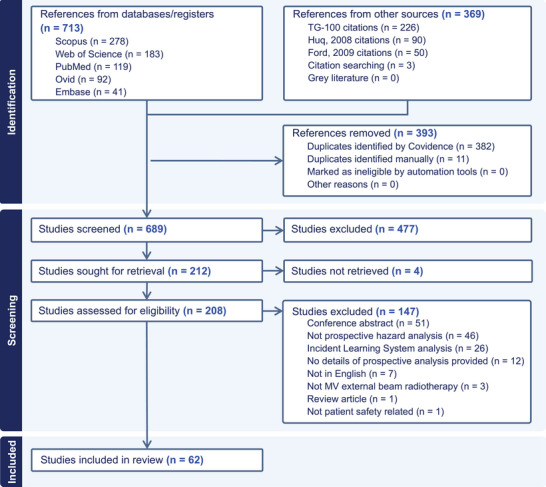
PRISMA flowchart for the systematic literature review.

From the raw searches undertaken in December 2023 and repeated in July 2024, 689 unique records were identified. 477 were excluded during title and abstract review, along with four studies after the full text was unable to be located. 151 studies were excluded during full text review, leaving 62 studies for inclusion in the review. The PRISMA^11^ flowchart is shown in Figure 1.

### 3.2 Overview of studies

**FIGURE 2:**
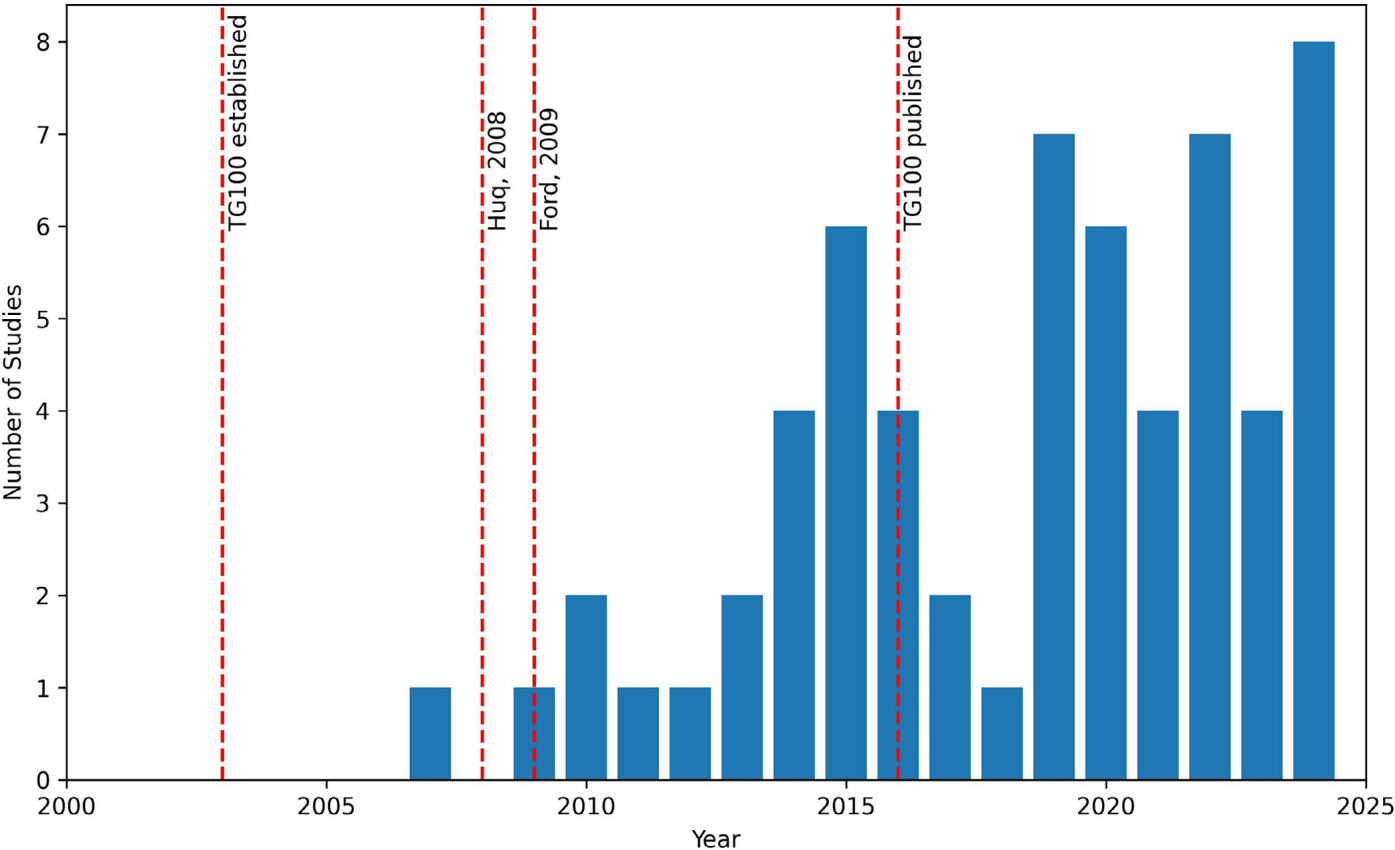
Publications per year for studies identified in the literature review (bar graph), with additional prospective hazard analysis in radiation therapy milestone dates given (dashed lines).

**FIGURE 3:**
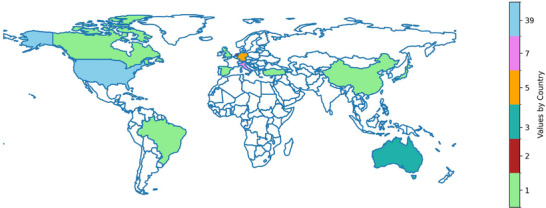
Geographical distribution of studies.

Table 2 provides an overview of the included studies and the focus of the analysis, Figure 2 shows the number of studies published per year since 2000, and Figure 3 details the geographic distribution of studies around the world. For systems included under c‐arm Linac or generic treatment pathways, the choice was made to separate out those studies where the primary focus was on the events required for the treatment of patients, which for the most part, ignored existing chart check and QA processes. This decision partly stemmed from TG‐100′s recommendation that no QA be considered when performing a risk analysis and partly due because studies that investigated the risk in checks were structured differently from studies that investigated the risk involved in the steps required to treat a patient.

**TABLE 2:** Summary of studies included in the review.

System	Subsystem	Count
C‐arm Linac or generic treatment pathways (Table 3)	Patient journey	17
	SRS	8
	SBRT	5
	Chart review	3
	TPS commissioning	1
	Software tool	1
	MRI‐only planning	1
	Surface image guidance	1
	VMAT total body irradiation	1
Halcyon/Ethos (Table 4)	Ethos	5
	Halcyon	2
	Commissioning	1
Tomotherapy (Table 5)	Tomo	4
	Rapid treatment	1
	Total marrow irradiation	1
MRIgRT (Table 6)	MRIdian	3
	Unity MRL	1
Other (Table 7)	Radiation planning assistant	2
	MLC tracking	1
	Lattice RT	1
	RefleXion	1
	CK SBRT	1
	Ransomware attack	1

Tables 3, 4, 5, 6, 7 summarize the key hazard analysis details for the studies applicable to the treatment platform specified. To consistently categorize the method of describing the system for studies applying FMEA, ‘process map’ is used when studies match the format used in TG‐100, and ‘workflow’ is used when the study uses a flowchart version of a processes map (typically these are also coarser than recommended by TG‐100). For other analysis techniques, the description used in the study is replicated in the table. Not every PHA technique uses a rating system or a threshold for high‐risk designation, hence they are only included when used by a study. Mitigation strategies are also referred to as recommendations for reducing the risk rating of an identified hazard or hazards. Where the authors specified the number of mitigation strategies in the study text, that number was used regardless of whether they were all included in the manuscript or not. Where the authors don't specify a number, an estimate is made from the details included in the manuscript, hence there are several studies where there is a lower limit to the number of mitigation strategies identified.

**TABLE 3:** Studies focused on C‐arm treatment systems.

Lead author; Year, Country	System	Hazard analysis technique	Method of describing the system	Identified hazards	Ranking method	Method for identifying high‐risk hazards	No. of high‐risk hazards	No. of mitigation strategies
Ekaette; 2007; Canada^7^		FTA	Workflow	77 (process type) + 40 (infrastructure type)	Probabilistic (expert elicitation)	NA	NA	–
Ford; 2009; US^9^		FMEA	Process map	127	RPN (Ford *et al.*, 2009)	Top ranked (RPN > 75)	15	28
Scorsetti; 2010; Italy^25^		Modified FMEA	IDEF Ø	27	Criticality index (CI = O × S × D; O and D: 1–4; S: 1–5)	Medium: 24 < CI < 46 High: CI > 45	1	4
Terezakis; 2011; US^26^		FMEA	Process map	127 (1st), 159 (2nd)	RPN (Ford *et al.*, 2009)	Top ranked	>15	4
Santanam; 2013; US^27^		FMEA; Other: DMAIC	Workflow	11	RPN (TG‐100)	None specified	11	1
Denny; 2014; US^28^		FMEA	Process map	108	RPN (O × S × D; Likert scale with 5 [O, D] or 7 [S] points)	Grouping and discussion	5	5
Ford; 2014; US^29^		FMEA	Process map	52	RPN (TG‐100)	RPN > 150	4	4
Noel; 2014; US^30^		FMEA	Flow diagram	127	RPN (TG‐100)	RPN > 199	43	>9
Huq; 2016; US^6^		FMEA; FTA	Process map	216	RPN (TG‐100)	Top ranked 20% or *S* > 8	47 (RPN), 27 (severity)	>15
Frewen; 2018; Australia^31^		FMEA	Process map	173	RPN (O × S × D; 4‐point Likert scale)	Top ranked 20% (RPN > 98) + *S* > 7	20	18
Baehr; 2020; Germany^32^		FMEA	Process map	29	RPN (O × S × D)	RPN > 100	11	–
Silvis‐Cividjian; 2020; The Netherlands^33^		STPA	Control structure	142 UCA, only 10 CS included	NA	NA		7
Gilmore; 2021; UK^34^		FMEA	Process map	40	RPN (TG‐100) & SOD	Top ranked	10 (RPN) 10 (SOD)	0
Mancosu; 2021; Italy^35^		FMEA	IDEF Ø	67	RPN (O × S × D; 1–4 O & D, 1–5 S)	23 < RPN < 46: medium‐risk RPN > 45: high‐risk	0	11
Lohmann; 2022; Germany^36^		Modified FMEA	Workflow	38	Severity + probability risk matrix	High	16	50
Ozbay; 2022; Turkey^37^		FTA	Workflow	87 CUT‐SET	Probabilistic (expert judgement)	Top ranked	5	0
Wong; 2022; US^38^		STPA	Control structure	118 UCA, 267 CS	NA	NA	NA	0
Ford; 2020; US^39^	Chart review	FMEA	Process map	594	phase 1: O, S and D (1‐3 ranking) phase 2 (high‐risk only): RPN (TG‐100)	Top ranked 40% or S > medium (2.4 out of 3)	112 for EBRT initial review (46 with RPN > 100), 55 in EBRT weekly and end‐of‐treatment review	3
Rassiah; 2020; US^40^	Chart review	FMEA	Workflow	50	RPN (TG‐100)	RPN > 20 or *S* > 3	18	>1
Wexler; 2017; US^41^	Commissioning	FMEA	Workflow	Manual: 47 Automatic: 36	RPN (TG‐100)	Top ranked	10 (manual) 10 (automatic)	1
O'Connell; 2019; US^17^	In‐house software tool	Modified FMEA	Process map	61	RPN (TG‐100)	Unclear	>6	–
Kim; 2019; US^42^	MRI‐only treatment planning	FMEA; FTA	Process map	Common: 125 MRI‐only unique: 47 MRI+CT unique: 19	RPN (TG‐100) & SOD	RPN > 100 OR *S* > 6	Unique to MRI‐only: 15 Unique to MRI+CT: 6	>15
Dragojevic; 2024; US^43^	RO peer review	FMEA	Workflow	39	RPN (O × S × D)	RPN > 100	21	1
Perks; 2012; US^44^	SBRT	FMEA	Flow chart	Unclear	RPN (O×S×D; 5‐point Likert scale for O, S, and D)	RPN > 20 (*S* = 10)	10	>10
Yang; 2015; US^45^	SBRT	FMEA	Workflow	63	RPN (Ford et al., 2009)	RPN > 150 and/or *S* > 7	8	0
Rusu; 2020; US^46^	SBRT	FMEA; FTA	Workflow	102	RPN (TG‐100)	Top 10 RPN + *S* > 7	14	10
Lee; 2021; US^47^	SBRT	FMEA; FTA	Process map	73	RPN (Ford et al., 2009)	Top ranked 25%	10	3
Benavente; 2024; Spain^48^	SBRT	FMEA	Process map	232	RPN (O × S × D)	Top ranked	10	10
Bright; 2022; US^49^	SIG	FMEA	Process map	57 (165 causes)	RPN (TG‐100)	RPN > 92 OR Severity > 7	7	15
Masini; 2014; Italy^50^	SRS	FMEA	Process map	116	RPN (Modified TG‐100)	RPN > 125 or *S* > 6	4	5
Manger; 2015; US^51^	SRS	FMEA; FTA	Process map	167	RPN (TG‐100)	RPN > 100	25 (RPN > 100) + 5 (highest 5 SIG specific FMs)	11
Younge; 2015; US^52^	SRS	FMEA	Process tree	99	RPN (O × S × D, 10‐point scale)	RPN(mean) > 150 RPN(rater) > 200 Or S(rater) = 10	5 (RPN(mean) > 150), 16 (RPN(individual) > 200), 30 (S(individual) = 10)	>21
Pawlicki; 2016; US^53^	SRS	FMEA; STPA	Process map; Control Structure	132 FMs; 83 UCA, 472 CS	RPN (Ford et al., 2009); NA	RPN > 300; NA	8; NA	0
Rah; 2016; US^54^	SRS	FMEA; Other: m‐HFMEA	Process map	167	RPN (TG‐100) [FMEA]; Risk matrix [m‐HFMEA]	RPN > 125 (FMEA); High or V. High (m‐HFMEA)	17 (FMEA); 14 (m‐HFMEA)	2
Teixeira; 2016; Brazil^55^	SRS	FMEA; FTA	Process map	Site A: 135, Site B: 104, Site C: 146	RPN (TG‐100)	RPN > 99	Site A: 22, Site B: 115, Site C: 110	–
Schuller; 2017; US^56^	SRS	FMEA	Process map	409	RPN (TG‐100)	RPN > 100 or *S* > 8	106	>10
Ralston; 2020; Australia^22^	SRS	Other: RABBIT	Workflow	14	Risk matrix	Medium or high	5	>5
Gray; 2023; US^57^	VMAT TBI	FMEA; FTA	Process map	128	RPN (TG‐100)	Top ranked 20%	27	6

Abbreviations: CS, causal scenarios; CUT‐SET, Set of basic events whose simultaneous occurrence ensures the top event occurs; D, detectability; DMAIC, define, measure, analyze, improve, and control; EBRT, external beam radiation therapy; FMEA, failure mode and effects analysis; FMs, failure modes; IDEF Ø, integration definition for function modelling; m‐HFMEA, modified healthcare FMEA; MS, mitigation strategies; MRI, magnetic resonance imaging; NS, not specified; O, occurrence; RABBIT, risk and benefit balance impact template; RO, radiation oncology; RPN, risk priority number; S, severity; SAPERO, patient safety, advanced techniques and for the assessment of the risk of unwanted events within the care pathway in the Radiotherapy sector; SBRT, stereotactic body radiotherapy; SIG, surface image guidance; SOD, 3‐digit composite number made up of S, O, and D; SRS, stereotactic radiosurgery; STPA, system theoretic process analysis; TBI, total body irradiation; UCA, unsafe control actions; US, United States; VMAT, volumetric modulated arc therapy.

**TABLE 4:** Studies focused on Halcyon/Ethos treatment systems.

Lead author; Year, Country	System	Hazard analysis technique	Method of describing the system	Identified hazards	Ranking method	Method for identifying high‐risk hazards	No. of high‐risk hazards	No. of mitigation strategies
Teo; 2019; US and Chang; 2022; US^18^, ^58^	Commissioning	FMEA and fuzzy FMEA	Process map	88	RPN (TG‐100) & Fuzzy RPN	Top ranked	5 for acceptance 10 for commissioning 5 for Fuzzy (commissioning SP 1–8)	15
Pawlicki; 2019; US^59^	Halcyon	STPA	Control structure	144 UCA, 385 CS	NA	NA	NA	–
Wegener; 2022; Germany^60^	Ethos	FMEA	Process Map	122	RPN (O × S × D; 5 point scale)	Top ranked	20	20
Wong; 2022; US^38^	Halcyon; Ethos	STPA	Control structure	Halcyon: 110 UCA, 254 CS Ethos: 119 UCA, 267 CS	NA	NA	NA	0
Wang; 2024; US^61^	Ethos	FMEA	Workflow	Not specified	RPN (O× S × D)	Not specified	20	∼10
Wegener; 2024; Germany^62^	Ethos	FMEA	Workflow	111	RPN (O × S × D; 10 point scale)	Top ranked	20	17
Wong; 2024; US, Australia, Netherlands^63^	Ethos	STPA	Control structure	18 UCA, 97 CS	NA	NA	NA	>97

Abbreviations: CS, causal scenarios; D, detectability; FMEA, failure mode and effects analysis; FMs, failure modes; MS, mitigation strategies; O, occurrence; RPN, risk priority number; S, severity; UCA, unsafe control actions; US, United States.

**TABLE 5:** Studies focused on TomoTherapy treatment systems.

Lead author; Year, Country	System	Hazard analysis technique	Method of describing the system	Identified hazards	Ranking method	Method for identifying high‐risk hazards	Number of high‐risk hazards	No. of mitigation strategies
Broggi; 2013; Italy^64^	TomoTherapy	FMEA	Process Map	74	RPN (TG‐100)	RPN > 80	21	6
Jones; 2015; US^65^	STAT RAD	FMEA	Process map	72	RPN (TG‐100)	RPN > 125	22	27
Broggi; 2015; Italy^66^	TomoTherapy	FMEA	Process map	30	RPN (TG‐100)	RPN > 80	9	8
Yamaguchi; 2019; US^67^	TomoTherapy	STPA	Control structure	99 UCA, only 1 CS included	NA	N/A	NA	19
Shen; 2019; China^68^	Total marrow irradiation	FMEA; FTA	Process map	122	RPN (TG‐100)	Top ranked 20%	Initial: 25; repeat: 20 (all new)	5
Giardina; 2023; Italy^23^	TomoTherapy	Other: SAPERO	Hierarchical task analysis	?	RPN (O×S×D) + criticality (FMECA)	Not specified	7	7

CS, causal scenarios; D, detectability; FMEA, failure mode and effects analysis; FMs, failure modes; MS, mitigation strategies; O, occurrence; RPN, risk priority number; S, severity; SAPERO, patient safety: advanced techniques and for the assessment of the risk of unwanted events within the care pathway in the Radiotherapy sector; STPA, system theoretic process analysis; UCA, unsafe control actions; US, United States.

**TABLE 6:** Studies focused on magnetic resonance imaging‐guided radiation therapy treatment systems (MRIgRT).

Lead author; Year, Country	System	Hazard analysis technique	Method of describing the system	Identified hazards	Ranking method	Method for identifying high‐risk hazards	Number of high‐risk hazards	No. of mitigation strategies
Kluter; 2021; Germany^12^	MRIdian	Modified FMEA	Process tree	89	Severity + probability risk matrix	Risk level 3	59	>59
Nishioka; 2022; Japan^69^	MRIdian	FMEA	Process map	153 (51 related to MRIgRT, 63 to MRIg online ART, & 66 to conventional RT)	RPN (TG‐100)	Top ranked 20% or *S* > 7	49 (31 due to RPN, 28 due to S, 10 due to both RPN & S)	144
Liang; 2023; US^70^	Unity MRL	FMEA	Process map	216	RPN (O × S × D; ranking: 1–5)	Top ranked 20% (average RPN, median RPN or S)	45	65
Behzadipour; 2024; US^71^	MRIdian	FMEA	Process map	279	RPN (O × S × D)	top ranked 20% (RPN or S)	47	3

Abbreviations: D, detectability; FMEA, failure mode and effects analysis; FMs, failure modes; MRI, magnetic resonance imaging; O, occurrence; RPN, risk priority number; S, severity; US, United States.

**TABLE 7:** Studies focused on other treatment systems.

Lead author; Year, Country	System	Hazard analysis technique	Method of describing the system	Identified hazards	Ranking method	Method for identifying high‐risk hazards	Number of high‐risk hazards	No. of mitigation strategies
Sawant; 2010; US^72^	MLC tracking	FMEA	Workflow	7	RPN (TG‐100)	RPN > 125	1	1
Veronese; 2015; Italy^73^	Cyberknife	FMEA	Process map	not reported	RPN (O × S × D)	RPN > 79 or *S* > 8	19	NS
Kisling; 2019; US^74^	Radiation planning assistant	FMEA	Process map	68 (with 113 potential causes)	RPN (TG‐100)	RPN > 200 OR *S* > 8	37	3
Nealon; 2022; US^75^	Radiation planning assistant	FMEA; FTA	Process map	290 (126 specific to RPA workflow, 164 non‐specific to RPA workflow)	RPN (TG‐100)	RPA specific & (RPN > 125 or *S* > 7)	21	>21
Simiele; 2023; US^76^	RefleXion	FMEA; Other: DMAIC	Workflow	100 physics plan check elements	RPN (TG‐100)	Top ranked 10 or *S* > 5	10 (RPN), 10 (severity)	2
Deufel; 2024; US^77^	Lattice radiation therapy	FMEA	Process map	12	RPN (TG‐100)	Top ranked	4	3
Vinogradskiy; 2024; US^78^	Ransomware attack	FMEA	Workflow	33	RPN (O × S × D)	Increase in RPN due to ransomware	NA	1

Abbreviations: D, detectability; DMAIC, define, measure, analyze, improve and control (aka 6‐Sigma); FMEA, failure mode and effects analysis; FMs, failure modes; NS, not specified; O, occurrence; RPN, risk priority number; S, severity; US, United States.

**FIGURE 4:**
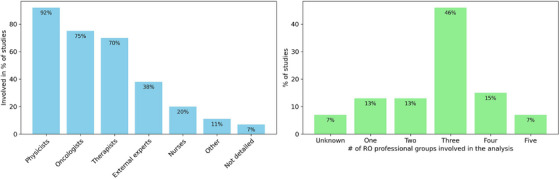
Percentage of studies each professional group is involved in (left), number of RO professional groups involved in each study (right).

Considering who was reported as undertaking the studies, the participants listed as being involved in the study were broken down into radiation oncology (RO) professional groups, external experts, and facilitators. RO professional groups include professions typically found in the RO department such as radiation oncologists, medical physics, radiation therapists, dosimetrists, and nurses. External experts are those whose professions are typically external to the RO department, and included engineers, quality control committee members, academics, software developers, administrators, vendor representatives, and analysts. Figure 4 details the percentage of studies each profession was a part of and how many RO professional groups were included in study teams. Of note is that the eight (13%) studies undertaken by a single RO professional group were undertaken by physicists.

The role of facilitator is one that some studies chose to include on the team undertaking the analysis and is typically filled by a person who has expertise performing the chosen PHA method. The purpose of the role is to guide the team performing the analysis so that they don't have to be experts in the method to use it. Facilitators were noted as being part of the team in 33% of studies. In six studies the facilitators were physicists, in three they were engineers, in three they were academics, in three they were noted as having experience in applying the chosen technique, in three they were from another institution or group, and in one they were from the main site (a study in which the analysis was conducted over three sites).

### 3.3 Prospective hazard analysis techniques

**FIGURE 5:**
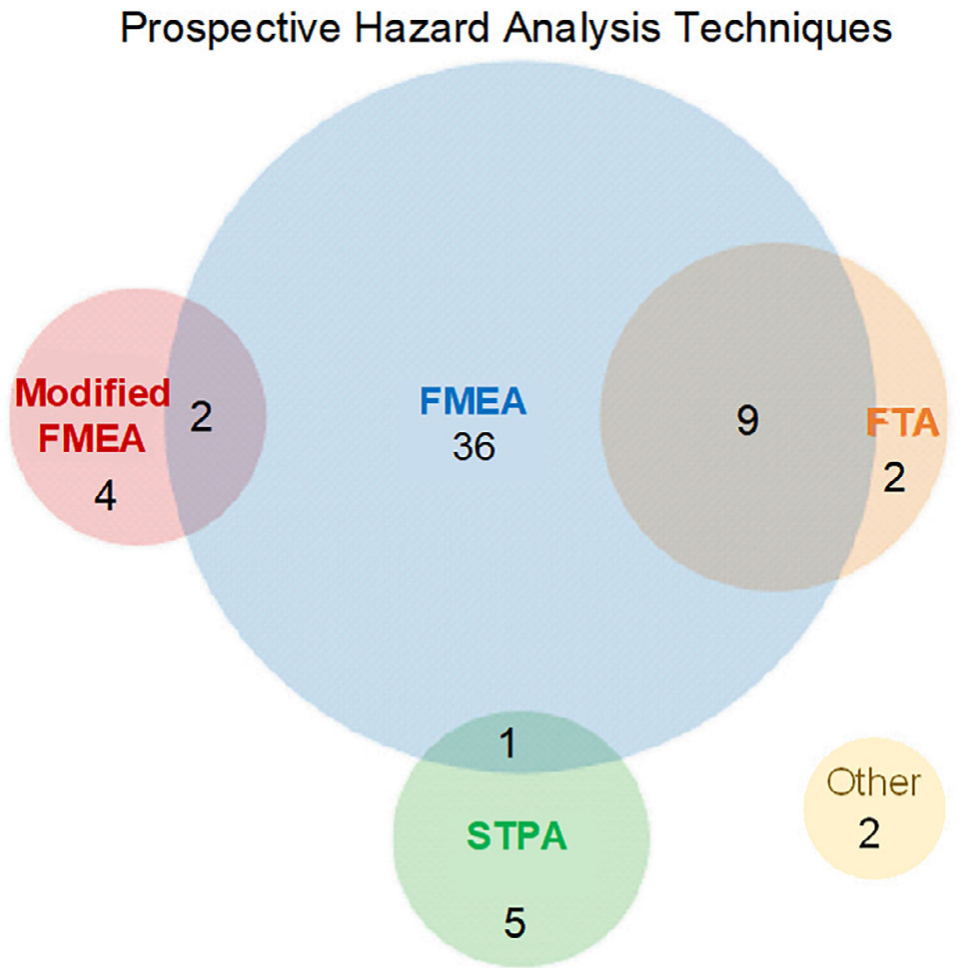
Venn diagram of prospective hazard analysis techniques used in the identified studies. FMEA = Failure mode and effect analysis, FTA = fault tree analysis, and STPA = system theoretic process analysis.

Across the 62 studies included in the review, 49 used a single analysis method while 12 used two analysis techniques (Figure 5).

#### 3.3.1 Failure mode and effects analysis (FMEA)

FMEA is one of the earliest developed PHA techniques, it is bottom‐up in nature, identifying all potential avenues of failure regardless of their impact or significance. FMEA^6^ involves generating a process map detailing the linear sequence of events involved in the process through analytical reduction, identifying the failure modes (FM) in each step of the process, assessing the effects of each FM in terms of their occurrence (O), detectability (D) and severity (S), and generating the risk priority number (RPN = O × S × D). Following generation of the RPN, a cut‐off is determined above which failure modes are identified as ‘high‐risk’ and require mitigation to reduce the risk, typically by reducing the occurrence or improving the detectability.

Most of the identified studies (48 of 62) used FMEA as described in TG‐100, some with slight modifications to the ranking method or in the way the system was described, but all identifiable as FMEA. Two of the studies that used FMEA, utilized it in the define, measure, and analyze steps of a 6‐sigma analysis (also known as DMAIC: define, measure, analyze, improve, and control) where the aim was to improve a specific process in the patient journey through radiation therapy.

Another six studies used FMEA derivatives:

‐ Process failure modes and effects and criticality analysis (PFMECA)^12^, ^13^
‐ Modified healthcare FMEA (m‐HFMEA)^14^, ^15^
‐ Failure modes and effects and criticality analysis (FMECA)^16^
‐ Software FMEA^17^
‐ Fuzzy failure modes and effects analysis (FFMEA)^18^

These derivatives have been developed to address one or more of the shortcomings of FMEA; PFMECA, m‐HFMEA, FFMEA, and FMECA were developed to change the way failures are ranked while software FMEA adapts FMEA to better assess the risks of software.

#### 3.3.2 System theoretic process analysis (STPA)

STPA is a technique developed by Leveson in the late 2000s that uses system theory to assess the hazards of a system.^19^, ^20^ Being top‐down in nature, it starts with identifying the events the assessment wants to prevent, then working through how they could come about, finally resulting in a list of scenarios that require assessment and mitigation. As STPA is based on system theory, the focus of the analysis is control loops and the flow of control actions and feedback within a system. Six of the included studies used STPA to assess a system for hazards.

#### 3.3.3 Fault tree analysis (FTA)

FTA (also known as probabilistic risk analysis, PRA) is a top‐down method of assessing the hazards in a system.^21^ Starting from an undesirable state (aka system failure), pathways of failure are identified interaction‐by‐interaction until an endpoint is reached. At each interaction, logic gates show the propagation of the error and how they contribute to the system failure. The inclusion of probabilities of failure at each interaction is where it gets its alternative name, probabilistic risk analysis. Of the included studies, 11 used fault tree analysis, however, only two studies used FTA alone, the rest of the studies used it alongside FMEA in the way described in TG‐100.

#### 3.3.4 Risk and benefit balance impact template

Developed in Australia in the late 2010s, the purpose of the risk and benefit balance impact template (RABBIT) is to perform an assessment of the risks and benefits introduced by a new technology or process prior to starting the work of introducing the technology or process.^22^ It is applied at a high level and helps to identify broad risks and to weigh them up against the benefits prior to committing time to introduce the change. One included study utilized RABBIT.

#### 3.3.5 SAPERO

SAPERO stands for patient safety: advanced techniques and for the assessment of the risk of unwanted events within the care pathway in the radiotherapy sector and was developed by Engineering Department of the University of Palermo and the Radiotherapy Department of ARNAS Civico.^23^ It is a hybrid method that combines hierarchical task analysis, cognitive task analysis, human error assessment and reduction technique, and fuzzy failure mode effects and criticality analysis. It was developed to combine the benefits of each of the hybridized methods and minimize the limitations, one included study utilized SAPERO.

### 3.4 High‐risk failure modes

As part of the data extraction from the studies, all studies involving the treatment of patients that identified failure modes and which included a threshold for designating high‐risk status, had the high‐risk failure modes extracted. Where available, the process step, failure mode, potential cause/s, ranking, and countermeasure were also extracted along with the article ID, target system and sub‐system. This extracted data is available as a spreadsheet in the Supporting Information. There was substantial variation in whether (and, if so, how) the studies included their identified failure modes; ranging from the inclusion of all identified failure modes to the inclusion of only a subset of the failure modes designated as high‐risk.

Of the 62 studies included in the analysis, 54 included a list of failure modes or ranked risks, 48 of those studies defined a threshold above which those failure modes would be ‘high‐risk’, while six either indicated which failure modes they defined as high‐risk or used a more complicated method of determining failure mode status. The studies that didn't include a list of failure modes were those that applied STPA (5) or FTA (2) alone, the remainder utilized a technique that generated failure modes or hazards that were ranked.

Scoring methods were dominated by risk priority number, a standard of most FMEA based techniques that is the multiplication of severity (S), occurrence (O), and detectability (D) (RPN = O × S × D). Methods of scoring O, S, and D varied across studies, the majority used scales of 1–10 based either on TG‐100^6^ or Ford et al.,^9^ Other methods included scales of 1–5, Likert scales and fuzzy scales. Other scoring methods included using a 2D risk matrix, whereby a rating is determined off severity and occurrence and creating a combination number made of SOD which effectively ranks according to severity.

The threshold required for a high‐risk rating is dependent on many factors including system, scale size (e.g. 1–10 vs. 1–5), scale definitions (what each value on the scale means) and the level of acceptable risk in a system. TG‐100 chose to consider the top 20%–25% of RPN and severity ranked failure modes but indicates that it is up to each individual team to determine the threshold appropriate for their system.

Going into more detail regarding choices of threshold, 26 studies used an RPN based threshold with 11 of these also specifying a severity threshold, the thresholds varied with RPN > 100 being the most common (six studies) and RPN > 125 the next most common (four studies). Of the remainder, 10 studies used a threshold RPN that was <100 and six used a threshold RPN that was ≥150. There were two studies that used an RPN threshold and a scale other than 1–10 for O, S, and D, both used a threshold of RPN > 45 with values of 1–4 for O and D and 1–5 for S. There were 20 studies that classified the top ranked failure modes as high‐risk: 11 used a set number (e.g. top 10) and nine used a percentage of the identified failure modes (e.g. top 20%). Six of these studies also defined a severity threshold above which failure modes would also be defined as high‐risk. The most common set number was 10 (six studies) and the most common percentage was 20% (seven studies). No study used only severity as the threshold for high‐risk failure modes, of the 16 studies that included a severity threshold, six studies used *S* > 7, four studies used *S* > 8, two studies used *S* = 10, two studies used *S* > 6 and the remaining two used *S* > 5 or *S* > 3.

Four studies used a rating matrix to determine failure mode status. In three studies the rating was determined using a combination of severity and occurrence. In one study, the rating was determined using likelihood and consequence of an identified risk. In all four studies, there were three possible ratings, equivalent to low, medium, and high.

A small number of studies had multiple thresholds, depending on whether they were considering the group average RPN or an individual's ranking. Additionally, many of the studies that were considering a specific technology either excluded any failure modes that didn't apply specifically to the technology in question or performed a secondary analysis of technology specific failure modes.

From the 54 studies, 579 failure modes were identified that are part of the patient journey (consult to end of treatment follow‐up excluding QA and chart check failures). A further 126 failure modes were identified that were a part of pre‐treatment chart checks and 35 failure modes that were a part of treatment chart checks. A complete list of the extracted failure modes is included in the Supporting Information.

Once all the high‐risk failure modes were extracted, each was assigned a step from a modified version of the TG‐100 process map (available in the Supporting Information) according to where the failure mode originated in the originating study. This was done to facilitate alignment, comparison, and grouping of failure modes across studies. Table 8 details the breakdown of the extracted failure modes into TG‐100 sub‐processes, where approximately half the high‐risk failure modes were associated with either initial or subsequent treatment of the patient (299), anatomy contouring (79), and treatment planning (72) are the next most populous sub‐processes. Of the 579 failure modes that were related to the patient journey, there were approximately 317 unique failure modes. In the Supporting Information, similar failure modes are indicated using the “Aligned with” column, where the ID # of the first similar failure mode is noted. For example, there were six failure modes identified more than 15 times across the included studies (note, the same high‐risk failure modes could be included multiple times in the one study). These high‐risk failure modes were dose prescription (25 occurrences), contouring (21), patient identification prior to treatment (20), image registration (16), patient motion during treatment (15), and motion monitoring (15).

**TABLE 8:** Failure modes per modified TG‐100 sub‐process from list of combined high‐risk failure modes according to origination of the failure mode.

# FMs from combined list	TG‐100 Sub‐process
299	Treatment (includes initial and subsequent)
79	Radiation treatment planning (RTP) anatomy contouring
72	Treatment planning
35	Initial treatment planning directive
26	CT simulation
19	Imaging and diagnosis
18	Plan preparation
13	Patient database information entered
11	Plan approval
7	Transfer images
2	Other imaging
2	Chart filling
0	Immobilization and positioning

Abbreviations: FM, failure modes, RTP, radiation treatment planning *Note*: failure modes may be assigned to multiple sub‐processes, e.g. initial and subsequent treatments.

Across all the high‐risk failure modes, 527 included a rating, of which 475 used a risk priority number (RPN) and 52 used a ranking (high or III based on a matrix). Of these 475 high‐risk failure modes that included an RPN, the majority (457) included values for occurrence (O), severity (S), and/or detection (D) while 18 solely reported the RPN. The methods for determining and reporting O, S, and D varied between studies, for example some discussed each FM until they got consensus, and others took a survey of all group members and analyzed the results. As mentioned previously, the scales and definitions used for O, S, and D also varied between studies.

Although the Supporting Information, where they were available in the original studies, includes the assigned values for occurrence (O), severity (S), and detection (D) along with the risk product number (RPN = O × S × D) they should not be used for any analysis. The reason for not using another site's O, S, or D is due to both the variation in methodology described above, the non‐deterministic nature of severity, the uncertainty in occurrence and the dependence of both detection and occurrence on process. As these factors vary between sites, the RPN should not be compared across sites.

### 3.5 Mitigation strategies

**FIGURE 6:**
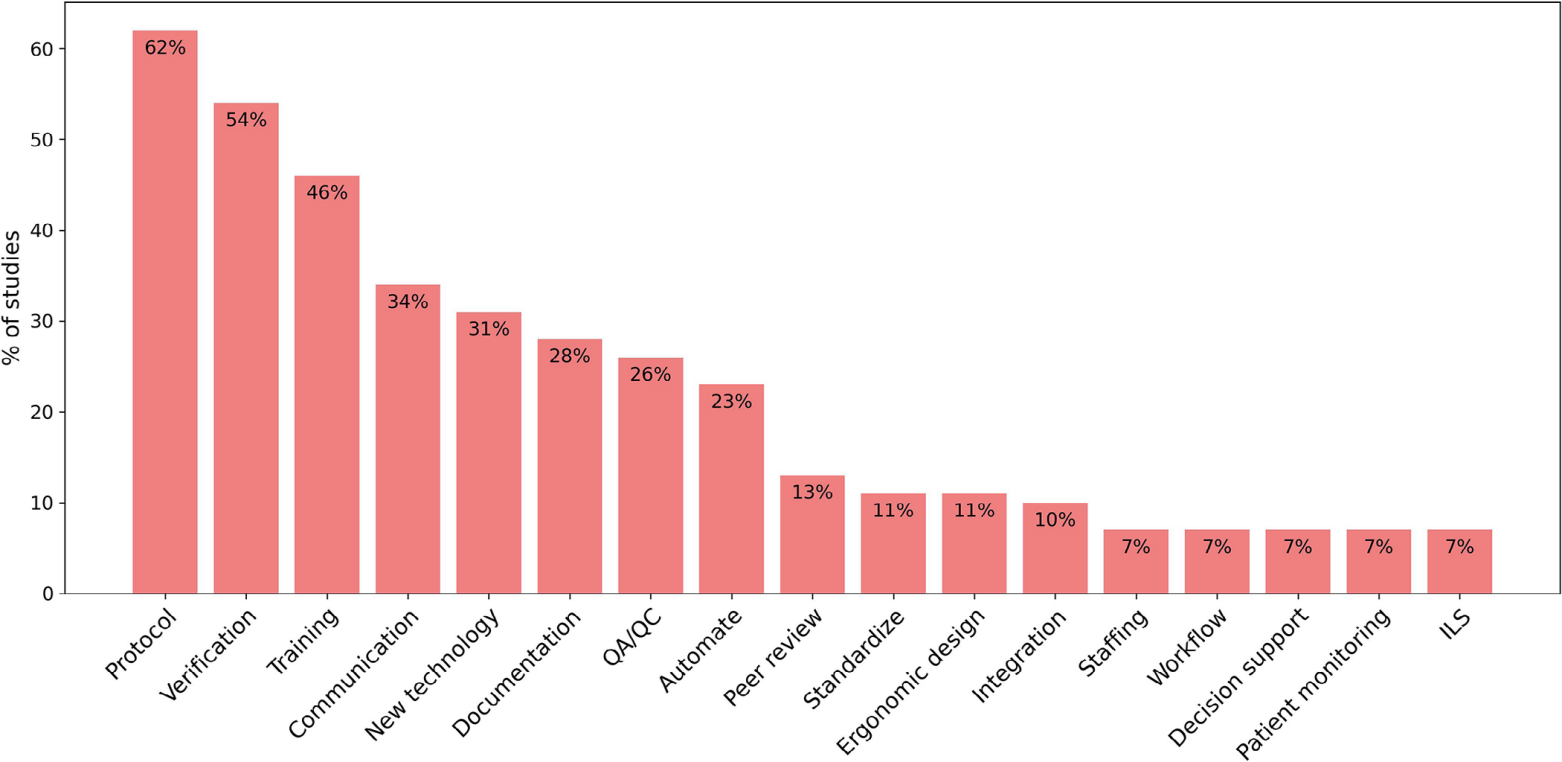
Summary of mitigation strategies recommended by studies. ILS = Incident learning system.

From each study that included mitigation strategies, a summary of the recommendations was made and then categorized. For example, where a study recommended new or modified work processes to reduce the risk of a hazard, this was assigned ‘new or modified protocol/process’. The naming categories were aligned and the number of studies that included each category counted, with the frequency distribution shown in Figure 6.

Of the mitigation strategies recommended by studies, the top four most frequent recommendations were those related to process, procedure or protocol (62%) followed by checks (54%) (including checklists, 2nd checks, etc.), training (44%) and communication (34%) (Figure 6). Further down the list of frequent mentions were new technologies, documentation, QA, and automation. A common factor between many of the proposed mitigation strategies were that they are low on the hierarchy of effective interventions^24^ and/or would have the effect of increasing workload rather than reducing it.

## 4 DISCUSSION

The authors have undertaken a systematic review of the literature for PHA studies involving x‐ray external beam radiation therapy. Of the 689 unique studies identified, 62 were determined to be eligible for inclusion. The purpose of the review was to determine what techniques and technologies have been assessed, how they have been assessed and what can be learnt from the published literature. The list of delivery systems and technologies assessed by PHA is included in Tables 3, 4, 5, 6, 7. Although the comparison of results between studies, be that failure modes or specific mitigation strategies, was an initial aim of this review, when the results were collated, it was found that there was minimal overlap between the studies on which comparisons could be made. The collation of high‐risk failure modes in the Supporting Information was determined to be the best method of comparing studies that included high‐risk failure modes.

One of the most notable findings of the review is that, of the mitigation strategies recommended by the studies, most of them lead to an increase in departmental load rather than its reduction. It is hypothesized that the reason for the increased departmental load is that the mitigation solutions focus on either improving detection of any error or attempting to increase reliability in the process so that errors occur less frequently rather than removing the source of the error. Very few studies, in their mitigation strategies, proposed a redesign of the process to minimize or remove the risk of a failure, meaning that the proposed mitigation strategies were in addition to the existing processes. This is likely because it is easier and quicker to add checks or improve reliability than it is to redesign a process to address the source of a failure mode. Additionally, if the source of a failure mode is in the hardware or software system, then modifying the process to mitigate the failure mode would likely require changes to the hardware or software which is out of the control of the site and thus not a reasonable option. In circumstances like these, the responsibility to mitigate the failure mode must sit with the vendor of the system.

It is worth noting that the increase in workload due to PHA is in opposition to the intent of TG‐100, where the aim was to find efficiencies by removing ineffective or insufficient tests and focusing efforts where they have the greatest impact. In the final report of TG‐100,^6^ in Sections 2 and 3.B the authors discuss the impacts on workforce resources that are resulting from the introduction of new technologies into patient treatment. Since then, there have been further technological developments, such as the clinical introduction of online and real‐time adaptive therapy and the accelerated incorporation of artificial intelligence into the treatment planning workflow. One reason for introducing prospective hazard analysis to the broader medical physics community was to enable individual sites to create a QA program that suited their technology mix, was cost effective and allowed them to manage their QA burden. What this review found was that managing the QA burden was a low or non‐existent priority for the included studies.

Many published consensus guidelines assessing QA needs in radiation therapy focus in detail on the mechanical properties of the treatment equipment. However, there hasn't been that same attention paid to plan preparation and associated processes nor to the processes that are involved in delivering treatment fractions. What many of the identified studies focused on was the human side of processes, hence the soft skills focus of the recommendations detailed above. Based on this review, it does not appear that the recommendations of TG‐100 alone are adequate to deal with the increasing QA burden of sites which suggests more research needs to be done to assess how to effectively manage the QA burden.

The rationale for performing PHAs has changed over the years. In the 2000s, and up to the release of the TG‐100 report in 2016, PHAs were focused on assessing existing techniques implemented at the study site to look for efficiencies and gaps in existing processes. After the publication of AAPM TG‐100 in 2016, the subject focus of PHAs has shifted toward new or emerging treatment systems or technologies and how to integrate them into the clinical workflow. The shift in PHA focus appears to largely be a result of the increased accessibility of advanced techniques starting in the late 2010s/early 2020s with, for example, the introduction of online adaptive radiation therapy, via systems such as Ethos, MRIdian and Unity MRI‐Linacs. This shift also appears to reflect the fact that existing consensus guidelines, such as the report of AAPM Task Group 142^79^ do not cover the full range of capabilities of these systems.

This lack of consensus guidance for such systems is understandable, as there must first be enough experience with a system to arrive at a consensus, and enough users of the system, for the consensus to be worthwhile for the wider community. However, this also means that early adopters of such systems who want to implement a quality management (QM) program are required to use PHA even if they are not experienced in the use of PHA.

### 4.1 Recommendations

#### 4.1.1 Resources for undertaking a PHA

Three of the included studies included some detail describing how they conducted the analysis. TG‐100^6^ provides a detailed description of how to perform a FMEA, with a useful example. Readers who are looking to perform a FMEA PHA would be strongly encouraged to read and follow the recommendations of that study. To simplify the ranking process, there are two studies^31^, ^32^ that describe alternative methods for ranking and obtaining a consensus amongst participants. Both mostly follow the TG‐100 process apart from the ranking method. Many of the included studies note that the ranking process's primary role is to qualitatively compare failure modes, thus changing the method of ranking does not detract from the overall hazard analysis.

For readers looking to use alternative hazard analysis techniques to FMEA, there were three main options identified: STPA, SAPERO, and RABBIT. Of these STPA is the most developed and is a substantial deviation from FMEA as it is based on a system theory‐based model instead of linear causation‐based models. The differing theoretical underpinning means the two methods focus on distinct aspects of risk and can identify different causes and mitigation strategies. There is a STPA handbook^20^ published in the mid‐2010s following the development of the underlying accident model (STAMP^19^) in the mid‐2000s and the process is still under active development. Further, there are six studies identified in this review that use STPA within radiation oncology that can be used for reference.

The SAPERO method is discussed by Giardina et al.,^23^ however, there doesn't appear to be any other studies that use the method and an internet search only returns non‐English results. The RABBIT^22^ method is a high‐level assessment of risk, intended to be performed prior to the purchase or consideration of new technologies or techniques. It provides initial useful information when deciding whether to pursue a project but does not provide insight into any specific risks in a process.

Studies by Nishioka et al.^80^ and Kornek and Bert^13^ both contain Supporting Information (spreadsheets, instructions) that may assist a reader in conducting an FMEA analysis of a process. The study by Nishioka et al. includes a google sheet designed to facilitate collaboration when ranking failure modes. While the study by Kornek and Bert includes a spreadsheet with an example process map, failure modes, and facilitates the ranking of the failure modes.

#### 4.1.2 Recommendations resulting from the analysis

The review found a lack of consistency in reporting of methods, results and outcomes published by PHA studies. The first recommendation of this review is for authors to include the full PHA analysis either as Supporting Information or to be available upon request if there are concerns the analysis might be misused. For an FMEA analysis, it would include the complete process map of the system, all the identified failure modes along with the ranking, the designated high‐risk threshold, the mitigation strategies recommended for each of the high‐risk failure modes, and the expected ranking following implementation. Given all these steps should be done as part of an FMEA process, making it available following publication shouldn't be an impediment. Additionally, it is recommended that those looking to perform PHA follow standardized procedures, examples include ISO14971:2019,^81^ TG‐100,^6^ STPA,^20^ IEC 60812,^82^ and MIL‐STD‐1629A.^16^

The rationale behind undertaking a PHA and the subsequent impact was not clearly reported in many studies. This lack of a rationale makes it difficult for readers to judge whether the process was successful or not and therefore whether to undertake one themselves. The second recommendation of this review is that authors publishing PHA should provide a high‐level interpretation of the original rationale behind the study (for example to fulfil a legislative requirement, to assess the safety of a new technology, and to assess patient throughput), what was achieved and what (if anything) would be done differently next time.

No study identified in this review discussed the removal of mitigation strategies targeting low ranking failure modes. A consequence of only adding mitigation strategies, is that the institutions performing the analysis would have seen an increase in their QA burden and leads to the question: why don't studies recommend removing mitigation strategies targeting low ranking failure mode? There are two potential answers that stem from the studies identified: (a) PHAs include the current QA in the PHA, or (b) the PHAs being performed either aren't detailed enough to identify all the potential failure modes. Considering (a), when studies assume the current QA in their PHA, they lose the ability to remove mitigation strategies because they are integral in the analysis. That is, their impact is unassessed, so the impact of removal cannot be determined and thus the *status quo* must remain. Considering (b), studies that aren't detailed enough are only able to provide insight into overarching failure modes because of combining multiple steps into a single larger step. For example, the TG‐100 process map steps: ‘setup fields’ and ‘import and fuse images’, both involve multiple smaller steps that are critical to achieve a safe output but are rarely included in an analysis. The result is less specific failure modes that focus on more consequential failures, and an analysis with a limited ability to remove mitigation strategies because the analysis isn't detailed enough to determine whether they are necessary. Note that less specific failure modes can also lead to overlooking failure modes that result from details as small as a mis‐click or an incorrect keyboard input, for example the Therac‐25^83^, ^84^ accidents. The third recommendation of this review is for more research to be undertaken to understand why PHA studies don't appear to be resulting in the rationalization of mitigation strategies and instead appear to be increasing the burden on departments.

## 5 CONCLUSION

62 studies were identified as performing a prospective hazard analysis in x‐ray external beam radiation therapy. The patient journey was the most analyzed process, other processes analyzed included SRS, SBRT, VMAT, surface guided, online adaptive RT, and MRI–guided RT. FMEA and its derivatives were the most common hazard analysis technique used by studies and from an analysis of the failure modes identified by these studies the greatest source of high‐risk failure modes was the delivery of patient treatment. For future publications of prospective hazard analysis studies, the authors recommend the entirety of the analysis is made available to readers, standardized protocols for the conducting of hazard analysis be followed, and the rationale behind the analysis be discussed alongside the outcomes of the analysis.

## CONFLICT OF INTEREST STATEMENT

The authors declare no conflict of interest.

## Supporting information



Supporting Information
